# Epidemiological analysis of respiratory and intestinal infectious diseases in three counties of Sichuan: the baseline survey of Disaster Mitigation Demonstration Area in western China

**DOI:** 10.7717/peerj.7341

**Published:** 2019-07-23

**Authors:** Zhiqiang Xue, Zhenbo Yang, Hui Sun, Jinghuan Ren, Mengzi Sun, Jiagen Li, Anning Zhang, Pingping Zheng, Pan Pan, Jing Dou, Li Shen, Yang Chen, Kexin Li, Tianyu Feng, Yaogai Lv, Chunli Bi, Lina Jin, Zhe Wang, Yan Yao

**Affiliations:** 1 Epidemiology and Biostatistics, School of Public Health, Jilin University, Changchun, China; 2 UNICEF Office for China, BeiJing, China; 3 Chinese Center for Disease Control and Prevention, BeiJing, China

**Keywords:** Respiratory infectious diseases, Intestinal infectious diseases, Distribution characteristics, Influencing factors, Principal component regression

## Abstract

**Background:**

Natural disasters can indirectly induce epidemics of infectious diseases through air and water pollution, accelerated pathogen reproduction, and population migration. This study aimed to explore the epidemiological characteristics of the main infectious diseases in Sichuan, a province with a high frequency of natural disasters.

**Methods:**

Data were collected from the local Centers for Disease Control infectious disease reports from Lu, Shifang and Yuexi counties from 2011 to 2015 and from the baseline survey of the Disaster Mitigation Demonstration Area in Western China in 2016. Principal component regression was used to explore the main influencing factors of respiratory infectious diseases (RIDs).

**Results:**

The incidence rates of RIDs and intestinal infectious diseases (IIDs) in 2015 were 78.99/100,000, 125.53/100,000, 190.32/100,000 and 51.70/100,000, 206.00/100,000, 69.16/100,000 in Lu, Shifang and Yuexi respectively. The incidence rates of pulmonary tuberculosis (TB) was the highest among RIDs in the three counties. The main IIDs in Lu and Shifang were hand-foot-mouth disease (HFMD) and other infectious diarrhea; however, the main IIDs in Yuexi was bacillary dysentery. The proportions of illiterate and ethnic minorities and per capita disposable income were the top three influencing factors of RIDs.

**Conclusions:**

TB was the key point of RIDs prevention among the three counties. The key preventable IIDs in Lu and Shifang were HFMD and other infectious diarrhea, and bacillary dysentery was the major IIDs in Yuexi. The incidence rates of RIDs was associated with the population composition, the economy and personal hygiene habits.

## Introduction

Outbreaks of infectious diseases usually occur after extreme weather and natural disaster events (McMichael, 2015a; McMichael, 2015b). These outbreaks may be due to large-scale population displacement and the increase in risk factors for disease transmission, such as lack of food, safe water, and sanitary toilets; poor personal hygiene and nutritional status, and unplanned and overcrowded sanctuaries (Ivers & Ryan, 2006; Kouadio et al., 2014). Haiti experienced a cholera outbreak in October 2010, approximately 10 months after the Haiti earthquake. In the following two years, 604,634 individuals were infected and 7,436 individuals died from cholera (Barzilay et al., 2013). In the three months after the 2008 Wenchuan earthquake in China, 2,414 cases of infectious diseases were reported in six affected counties. Among them, fever with respiratory abnormalities and diarrhea accounted for 86.21% of all symptoms (Zhang et al., 2012). A diarrhoeal outbreak broke out in Dhaka, Bangladesh, with more than 17,000 cases after the devastating floods of June 2004 (Qadri et al., 2005).

Sichuan is located in southwestern China and is characterized by overpopulation, less land, and a complex geography. Sichuan Province is in the transition zone between the Qinghai-Tibet Plateau and the Sichuan Basin; there are many minerals in this region, and the precipitation in Sichuan Province is abundant and concentrated, with the precipitation from May to October accounting for approximately 70% of the annual rainfall. Because of these factors, Sichuan Province is one of the most prone to suffer from natural disasters (Liao et al., 2016). On May 12, 2008, an earthquake of magnitude 8.0 occurred in Wenchuan. On April 20, 2013, an earthquake of magnitude 7.0 occurred at the junction of Lushan County and Tianquan County in Yaan City. On August 8, 2017, a magnitude 7.0 earthquake occurred in Jiuzhaigou. Hence, understanding the epidemiological characteristics of major infectious diseases in Sichuan may provide useful information that can be used for targeted prevention measures after environmental disasters.

The baseline survey of the Disaster Mitigation Demonstration Area in Western China aims to understand the health status of residents and the risk of natural disasters. The survey investigated the Lu, Shifang and Yuexi counties in Sichuan from March to October 2016. In this research, we analysed the spatial, temporal and population distributions of RIDs and IIDs in the three counties from 2011 to 2015. We also explored the influencing factors of RIDs by principal component regression to provide targeted prevention measures.

## Methods

### Subjects

The data of this study consisted of two parts. Part 1 included meteorological, disaster-related information, population composition and economic information for the three counties, health-related behavior and resident awareness levels. Part 2 included the incidence rates of infectious diseases in the three counties.

**Figure 1:**
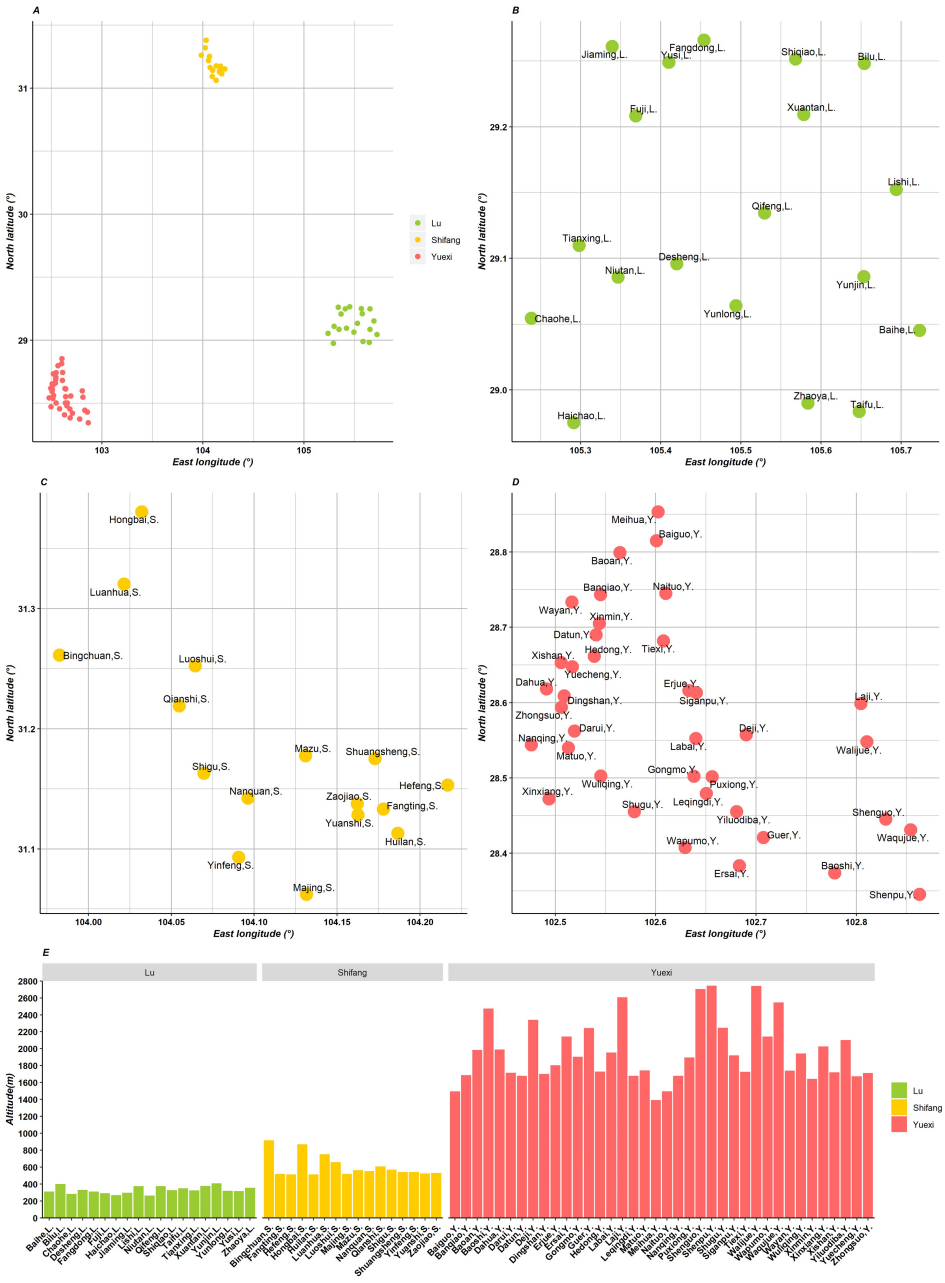
Geographic map of three counties in Sichuan, China. (A) The location of the three counties; (B, C, D) the coordinate of towns in Lu, Shifang and Yuexi, respectively; (E) the altitude histogram of the three counties.

Part 1: Data were derived from the baseline survey of the Disaster Mitigation Demonstration Area in Western China in 2016, which was completed by the Chinese Center for Disease Control and Prevention (Chinese CDC). We investigated the Lu, Shifang and Yuexi counties in Sichuan Province, which have different geographic social and economic features. Figure 1 shows the coordinates and altitudes of the towns in Lu, Shifang and Yuexi. Lu is located on the plains and hills, with an annual precipitation of 106.6 mm and annual mean temperature of 18.9 °C in 2015. No towns in Lu are located in an earthquake hazard zone. Shifang is located on the plains, with an annual precipitation of 765.4 mm and annual mean temperature of 17.3 °C in 2015; 66.7% of towns in Shifang are located in an earthquake hazard zone. Yuexi is located on the mountains, with an annual precipitation of 1215.1 mm and annual mean temperature of 14.1 °C in 2015. In Yuexi, 50.0% of towns are located in an earthquake hazard zone (Table 1).

**Table 1:** Meteorological and disaster-related information of three counties in Sichuan, China.

	Lu					Shifang					Yuexi				
	2011	2012	2013	2014	2015	2011	2012	2013	2014	2015	2011	2012	2013	2014	2015
Annual precipitation (mm)	47.0	103.5	75.3	112.4	106.6	707.7	674.4	1416.8	960.5	765.4	803.9	953.0	1021.8	1170.3	1215.1
Minimum temperature (°C)	−1.0	0.9	−0.7	0.1	2.7	−3.4	−3.0	−3.7	−2.0	−0.4	−6.3	−6.3	−6.2	−6.4	−5.5
Maximum temperature (°C)	41.4	38.9	40.1	38.8	37.4	35.5	36.1	36.9	34.6	35.9	37.5	34.2	35.7	33.1	34.5
Annual mean temperature (°C)	18.2	17.6	18.8	17.9	18.9	16.2	16.2	17.1	16.6	17.3	13.0	13.0	14.0	13.5	14.1
Frequency of natural disasters	3	1	3	1	2	0	3	5	0	0	0	3	4	8	8
Proportion of towns in an earthquake hazard zone (%)				0.0				66.7					50.0		
The contingency plan of flood, launched in 2011–2015				40				29					224		

Part 2: Data were derived from “the Infectious Disease Reporting Information Management System” in the National Legal Infectious Disease Reporting System from the Chinese CDC from 2011 to 2015.

### Data collection

Part 1: The data for demographic characteristics and meteorological and disaster-related information were derived from the local government. The monitoring capacity was derived from the CDC at the county level. Information on residents’ health-related behaviors and awareness of health-related knowledge was collected through epidemiological field surveys. The questionnaire was designed by the Health Emergency Center of the China CDC. The CDC trained investigators in the three counties, and they conducted the investigations. In each of the three counties, towns were handled as units, and each town randomly selected one village and randomly selected 50 residents from the extracted villages to conduct household surveys. The planned sample size was 2250, and 2237 valid samples were obtained (989 in Lu, 797 in Shifang, 451 in Yuexi), for a response rate of 99.4%.

Part 2: The cases of infectious diseases were reported by the local CDC through the “Infectious Disease Reporting Information Management System” from 2011 to 2015.

### Variable definition

The diagnostic criteria for each disease are listed in the File S1. According to the transmission route and the incidence rates of local infectious diseases, in our research RIDs included measles, pulmonary tuberculosis (TB), scarlet fever, mumps, rubella, varicella and pertussis; IIDs included hepatitis A, hepatitis E, bacillary dysentery, typhoid and paratyphoid, acute hemorrhagic conjunctivitis, other infectious diarrhea, hand-foot-mouth disease (HFMD), acute flaccid paralysis (AFP) and amebic dysentery.

### Statistical analysis

We used a heat map to explore the incidence rates of major RIDs and IIDs in each village and town from 2011 to 2015 using the “ggplot2” package. The *X*-axis represents the towns, the *Y*-axis represents the diseases from 2011 to 2015, and the colour represents the incidence rates.

Principal component regression was used to address multiple collinearities and research with more independent variables but insufficient sample size. First, we extracted information on independent variables by principal component analysis. Then, we took the incidence rates of RIDs as the dependent variable (*y*) and the three principal component (PC) scores as independent variables to build a linear regression model (by stepwise regression method) and obtained a standardized regression equation. Finally, we generated a standardized regression equation of the original variable through the transformation of the PC score.

The ratios are presented as the medians and inter-quartile ranges, which were compared using the Wilcoxon rank-sum test. We described the epidemic characteristics of infectious diseases through heat maps and histograms using the “ggplot2” package in R version 3.5.0 (Wickham, 2016; R Core Team, 2018). Statistical descriptions, principal component analysis, and linear regression were performed using SPSS 24.0 (IBM SPSS, IBM Corp, Armonk, NY, USA). Statistical significance was set at *P* < 0.05.

## Results

As Table 2 shows, the median of the proportion of individuals in towns in Yuexi younger than 5 years and older than 60 years was 19.66% (15.20%, 24.07%), which was lower than those in Lu and Shifang (*P* < 0.001). The median of the proportion of female in towns in Yuexi was 44.63% (36.09%, 47.85%), which was lower than those in other counties (*P* = 0.011). The median of the proportion of ethnic minorities in towns in Yuexi was 94.60% (48.32%, 100.00%), which was higher than those in Lu and Shifang. The median of per capita disposable income in towns in Lu was 19753.50 (12666.75, 21582.00) Yuan, which was higher than those in Shifang (14195.00 Yuan) and Yuexi (2822.50 Yuan). The proportions of residents who had adequate health-related behaviors and awareness of health-related knowledge in towns in Shifang were higher than those in other counties.

From 2011 to 2015, the frequencies of natural disasters in Lu, Shifang and Yuexi were 10, eight and 23, respectively, and the numbers of launched contingency plans for floods were 40, 29 and 224, respectively (Table 2). In Lu, Shifang and Yuexi, the numbers of water examination items for general testing were 34, 42 and seven, respectively, and for emergency testing, the numbers were two, seven and seven, respectively; the numbers of pathogenic microorganism examination items for general testing were 6, 10 and 3, respectively, and for emergency testing, the numbers were six, two and three, respectively (Table S1).

**Table 2:** Description of population composition, health related behaviours, and awareness of towns of three counties in Sichuan, China.

	Lu (1,077,301)			Shifang (434,958)			Yuexi (349,929)			*χ*^2^	*P-value*
	P_25_	P_50_	P_75_	P_25_	P_50_	P_75_	P_25_	P_50_	P_75_		
Proportion of population <5 years and >60 years (%)	23.70	26.22	29.86	26.32	27.63	30.03	15.20	19.66	24.07	21.976	<0.001
Proportion of female population (%)	46.64	48.29	49.71	39.91	49.43	52.16	36.09	44.63	47.85	8.953	0.011
Proportion of ethnic minorities population (%)	0.13	0.27	0.34	0.07	0.13	0.19	48.32	94.60	100.00	33.360	<0.001
Proportion of illiterate population (%)	1.37	4.18	6.66	0.00	3.72	6.35	0.96	9.68	29.15	7.969	0.019
Per capita disposable income (Yuan)	12666.75	19753.50	21582.00	14156.50	14195.00	14206.50	2009.00	2822.50	5322.50	50.316	<0.001
Proportion of correct drinking habits population (%)	38.61	51.96	62.38	57.01	62.73	74.25	0.00	0.00	0.00	67.629	<0.001
Proportion of correct hand-washing habits population (%)	19.42	28.57	41.29	55.00	62.13	66.00	0.00	0.00	0.00	53.221	<0.001
Proportion of population had the skill of CPR (%)	8.53	12.92	18.39	13.66	19.15	32.00	0.00	0.00	0.00	55.561	<0.001
Proportion of population used sanitary toilet (%)	68.37	80.28	85.56	89.35	96.25	100.00	0.00	0.00	0.00	31.103	<0.001
Awareness rate of infectious disease prevention and control knowledge (%)	0.00	0.00	0.00	0.00	0.00	8.12	0.00	0.00	0.00	13.343	0.001
Awareness rate of emergency telephone (%)	22.67	32.92	41.61	21.46	37.35	57.50	0.00	0.00	0.00	65.822	<0.001

**Notes.**

*P*_*x*_ percentile *x* CPR cardiopulmonary resuscitation

The confirmed diagnosis rates of RIDs from 2011 to 2015 were 43.56%, 16.25%, 23.23%, 24.26% and 29.78%, respectively, in Lu; 23.46%, 32.72%, 24.83% and 30.98%, respectively, in Shifang; and 32.67%, 29.37%, 31.82%, 43.05% and 18.32%, respectively, in Yuexi (Table 3). The confirmed diagnosis rates of IIDs from 2011 to 2015 were 33.59%, 41.58%, 24.57%, 22.22% and 32.97%, respectively, in Lu; 47.16%, 36.05%, 48.41% and 13.23%, respectively, in Shifang; and 62.04%, 66.86%, 58.89%, 40.44% and 38.91%, respectively, in Yuexi (Table 4).

**Table 3:** Diagnosis rate of respiratory infectious diseases of three counties in Sichuan, China, 2011–2015.

		Lu					Shifang^a^				Yuexi				
Year		2011	2012	2013	2014	2015	2011	2012	2013	2014	2011	2012	2013	2014	2015
Measles	Confirmed	1	1	1	1	0	2	0	0	1	26	0	2	5	52
	Total^b^	1	1	1	1	0	2	0	0	1	29	0	2	5	53
	C/T(%)	100.00	100.00	100.00	100.00	0.00	100.00	0.00	0.00	100.00	89.66	0.00	100.00	100.00	98.11
Pulmonary tuberculosis	Confirmed	476	159	211	154	177	130	137	109	113	153	157	145	122	67
	Total	883	731	738	536	531	334	323	323	304	422	393	393	268	566
	C/T(%)	53.91	21.75	28.59	28.73	33.33	38.92	42.41	33.75	37.17	36.26	39.95	36.90	45.52	11.84
Scarlet fever	Confirmed	0	0	0	0	0	0	0	1	0	0	0	0	0	0
	Total	2	6	6	3	1	3	3	9	3	0	1	1	0	2
	C/T(%)	0.00	0.00	0.00	0.00	0.00	0.00	0.00	11.11	0.00	0.00	0.00	0.00	0.00	0.00
Mumps	Confirmed	0	0	0	0	1	0	1	1	0	0	1	0	0	0
	Total	206	239	171	99	65	240	104	114	60	41	101	22	5	12
	C/T(%)	0.00	0.00	0.00	0.00	1.54	0.00	0.96	0.88	0.00	0.00	0.99	0.00	0.00	0.00
Rubella	Confirmed	0	3	1	0	0	5	4	1	0	2			3	3
	Total	3	26	1	0	0	5	4	5	0	34	4	0	3	3
	C/T(%)	0.00	11.54	100.00	0.00	0.00	100.00	100.00	20.00	0.00	5.88	0.00	0.00	100.00	100.00
Varicella	Confirmed	–	–	–	–	–	–	–	–	–	0	0	0	0	0
	Total	409	281	280	236	250	98	207	254	144	27	33	43	17	26
	C/T (%)	–	–	–	–	–	–	–	–	–	0.00	0.00	0.00	0.00	0.00
Pertussis	Confirmed	0	0	0	0	1	0	0	0	0	0	0	0	0	0
	Total	0	0	0	0	4	0	0	0	0	1	6	1	4	4
	C/T (%)	0.00	0.00	0.00	0.00	25.00	0.00	0.00	0.00	0.00	0.00	0.00	0.00	0.00	0.00
Total	Confirmed	477	163	213	155	179	137	142	112	114	181	158	147	130	122
	Total^c^	1,095	1003	917	639	601	584	434	451	368	554	538	462	302	666
	C/T (%)	43.56	16.25	23.23	24.26	29.78	23.46	32.72	24.83	30.98	32.67	29.37	31.82	43.05	18.32

**Notes.**

a Lack of diagnostic data for infectious diseases in 2015.

b Included clinical diagnosis cases, confirmed cases and suspected cases.

c Not included varicella in the total cases, because Lu and Shifang lacked the diagnostic categorical of varicella.

**Table 4:** Diagnosis rate of intestinal infectious diseases of three counties in Sichuan, China, 2011–2015.

		Lu					Shifang^a^				Yuexi				
Year		2011	2012	2013	2014	2015	2011	2012	2013	2014	2011	2012	2013	2014	2015
Hepatitis A	Confirmed	13	23	9	9	9	11	10	8	12	46	55	44	39	38
	Total^b^	27	26	15	11	9	11	10	10	12	48	55	44	39	38
	C/T (%)	48.15	88.46	60.00	81.82	100.00	100.00	100.00	80.00	100.00	95.83	100.00	100.00	100.00	100.00
Hepatitis E	Confirmed	34	27	10	18	15	1	3	5	1	3	3	0	1	3
	Total	34	27	11	18	15	1	4	5	1	3	3	0	1	3
	C/T (%)	100.00	100.00	90.91	100.00	100.00	100.00	75.00	100.00	100.00	100.00	100.00	0.00	100.00	100.00
Bacillary dysentery	Confirmed	9	4	5	1	0	1	20	2	3	145	167	86	53	39
	Total	20	10	13	15	1	5	27	2	3	228	239	153	107	123
	C/T (%)	45.00	40.00	38.46	6.67	0.00	20.00	74.07	100.00	100.00	63.60	69.87	56.21	49.53	31.71
Typhoid and paratyphoid	Confirmed	0	0	0	0	1	0	0	0	0	1	0	0	0	0
	Total	0	0	3	1	2	0	0	0	0	3	1	1	1	3
	C/T (%)	0.00	0.00	0.00	0.00	50.00	0.00	0.00	0.00	0.00	33.33	0.00	0.00	0.00	0.00
Acute hemorrhagic conjunctivitis	Confirmed	0	0	0	0	0	0	0	0	0	0	0	0	0	0
	Total	15	17	29	24	23	14	11	14	12	0	0	0	0	0
	C/T (%)	0.00	0.00	0.00	0.00	0.00	0.00	0.00	0.00	0.00	0.00	0.00	0.00	0.00	0.00
Other infectious diarrhea	Confirmed	18	18	15	26	76	223	199	305	112	6	11	18	12	10
	Total	102	106	132	194	276	277	214	317	121	32	44	46	36	33
	C/T (%)	17.65	16.98	11.36	13.40	27.54	80.51	92.99	96.21	92.56	18.75	25.00	39.13	33.33	30.30
Hand-foot-mouth disease	Confirmed	56	96	47	102	82	21	16	15	68	0	0	0	4	0
	Total	189	218	147	439	229	237	422	344	1333	10	11	7	85	36
	C/T (%)	29.63	44.04	31.97	23.23	35.81	8.86	3.79	4.36	5.10	0.00	0.00	0.00	4.71	0.00
Amebic dysentery	Confirmed	0	0	0	0	0	0	0	0	0	0	0	1	1	3
	Total	0	0	0	0	0	0	0	0	0	0	0	2	3	3
	C/T (%)	0.00	0.00	0.00	0.00	0.00	0.00	0.00	0.00	0.00	0.00	0.00	50.00	33.33	100.00
Total^c^	Confirmed	130	168	86	156	183	257	248	335	196	201	236	149	110	93
	Total	387	404	350	702	555	545	688	692	1482	324	353	253	272	239
	C/T (%)	33.59	41.58	24.57	22.22	32.97	47.16	36.05	48.41	13.23	62.04	66.86	58.89	40.44	38.91

**Notes.**

a Lack of diagnostic data for infectious diseases in 2015.

b Included clinical diagnosis cases, confirmed cases and suspected cases.

c Not included AFP in the total cases, because the three counties lacked the diagnostic categorical of AFP.

### Disease incidence rates composition and trend of RIDs and IIDs

**Figure 2:**
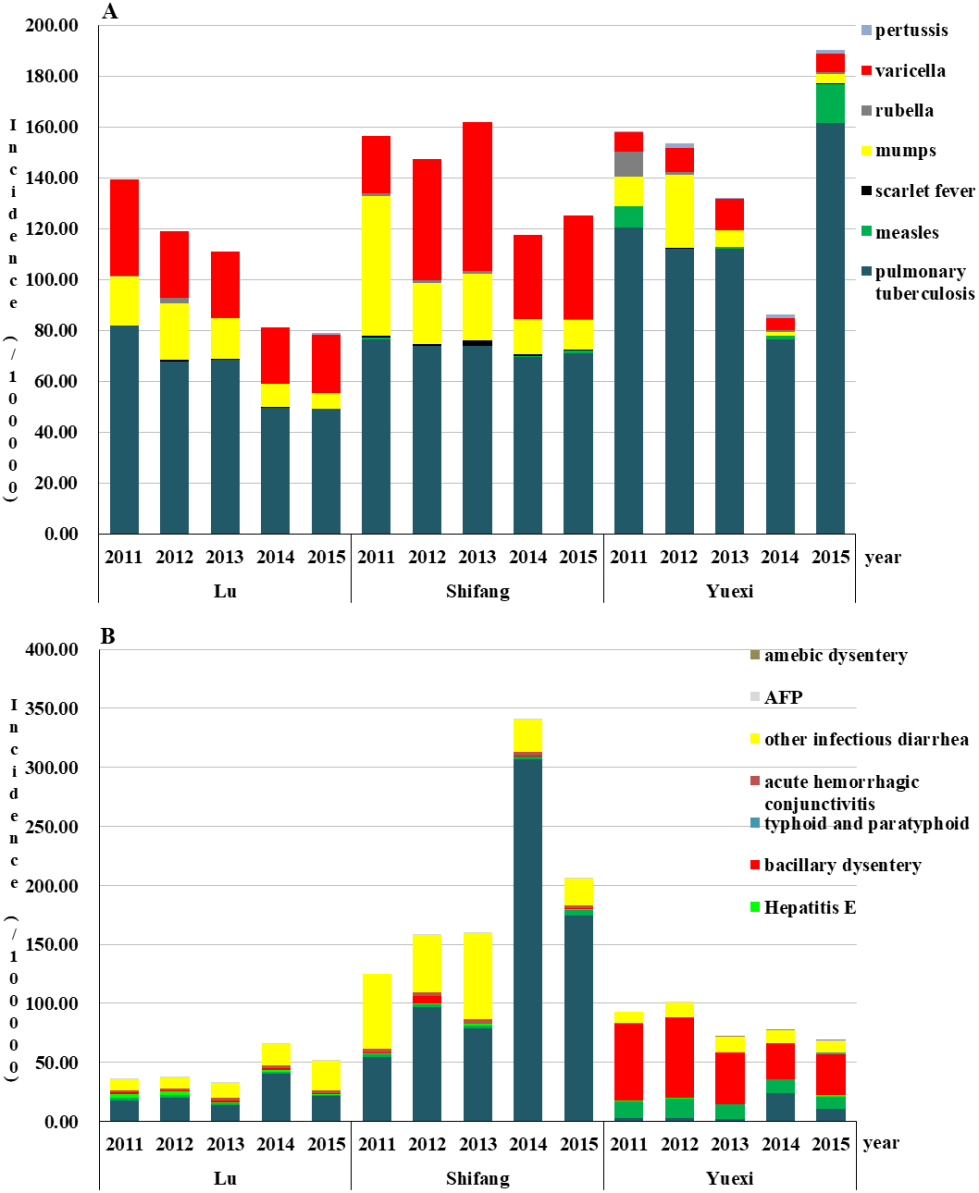
Incidence rates of respiratory (A) and intestinal (B) infectious diseases of three counties in Sichuan, China, 2011–2015.

The order of the incidence rates of RIDs from high to low was Yuexi, Shifang, and Lu, except for in 2013 and 2014. The incidence rates of RIDs in Lu showed a downward trend from 2011 to 2015; the highest incidence rates was 139.61/100,000 in 2011, and the lowest was 78.99/100,000 in 2015. The top three incidences of RIDs were TB, varicella, and mumps from 2011 to 2015. In Shifang, the highest incidence rates of RIDs was 162.08/100,000 in 2013, and the lowest was 117.71/100,000 in 2014; the top three incidences were TB, varicella, and mumps from 2011 to 2015 (were TB, mumps, and varicella in 2011). The incidence rates of RIDs in Yuexi showed a downward trend from 2011 to 2014; the highest incidence rates was 190.32/100,000 in 2015, and the lowest was 86.30/100,000 in 2014. The incidence rates of TB in Yuexi was highest than the other two regions in the five-year period (Fig. 2 and Table S2).

The order from high to low of the incidence rates of IIDs was Shifang, Yuexi, and Lu from 2011 to 2015. The incidence rates of IIDs in Lu showed an upward trend from 2011 to 2014; the highest incidence rates was 65.63/100,000 in 2014, and the lowest was 32.86/100,000 in 2013; the top three incidences were HFMD, other infectious diarrhea and hepatitis A (2011∼2012) and acute hemorrhagic conjunctivitis (2013∼2015). The incidence rates of IIDs in Shifang showed an upward trend from 2011 to 2014; the highest incidence rates was 344.95/100,000 in 2014, and the lowest was 120.30/100,000 in 2011. The top three incidences were HFMD (2012∼2015), other infectious diarrhea, acute hemorrhagic conjunctivitis (2013∼2015) and hepatitis A (2014∼2015). In Yuexi, the highest incidence rates of IIDs was 101.16/100,000 in 2012, and the lowest was 69.16/100,000 in 2015; the top three incidences were bacillary dysentery, hepatitis A and other infectious diarrhea (Fig. 2 and Table S3).

### Regional distribution of RIDs and IIDs

**Figure 3:**
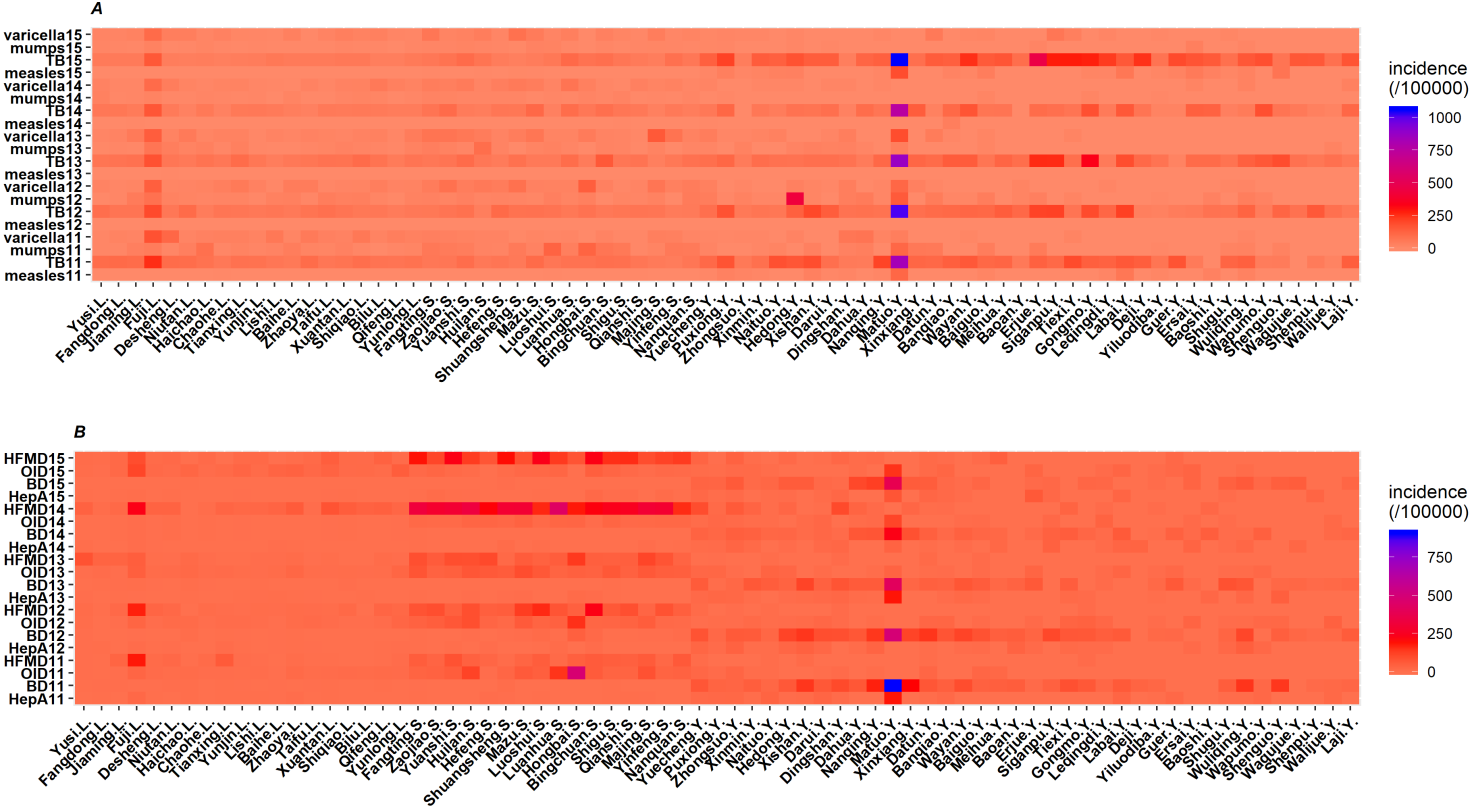
Heat map of incidence rates of respiratory (A) and intestinal (B) infectious diseases of three counties in Sichuan, China, 2011–2015. TB, pulmonary tuberculosis; HFMD, hand-foot-mouth disease; OID, other infectious diarrhea; BD, bacillary dysentery; HepA, hepatitis A; value is quadratic root transformation of incidence; .L, .S and .Y indicate that this village or town belongs to Lu, Shifang and Yuexi, respectively.

The heat map results illustrated that the distribution patterns of major RIDs were similar from 2011 to 2015, and the patterns between different villages and towns were similar in the same county. TB was the most prevalent RIDs in the three areas. The incidence rates of TB in Yuexi was higher than that in other areas, and the incidences of Matuo township were the highest from 2011 to 2015. The incidences of varicella and mumps in Lu and Shifang were generally higher than those in Yuexi (Fig. 3).

Like RIDs, the distribution patterns of IIDs were similar among different villages and towns in the same county from 2011 to 2015. However, the distribution patterns of major IIDs were different among the three counties. The major IIDs in Lu and Shifang were HFMD and other infectious diarrhea, while those in Yuexi were bacillary dysentery, hepatitis A and other infectious diarrhea (Fig. 3).

### Monthly incidence rates of RIDs and IIDs

**Figure 4:**
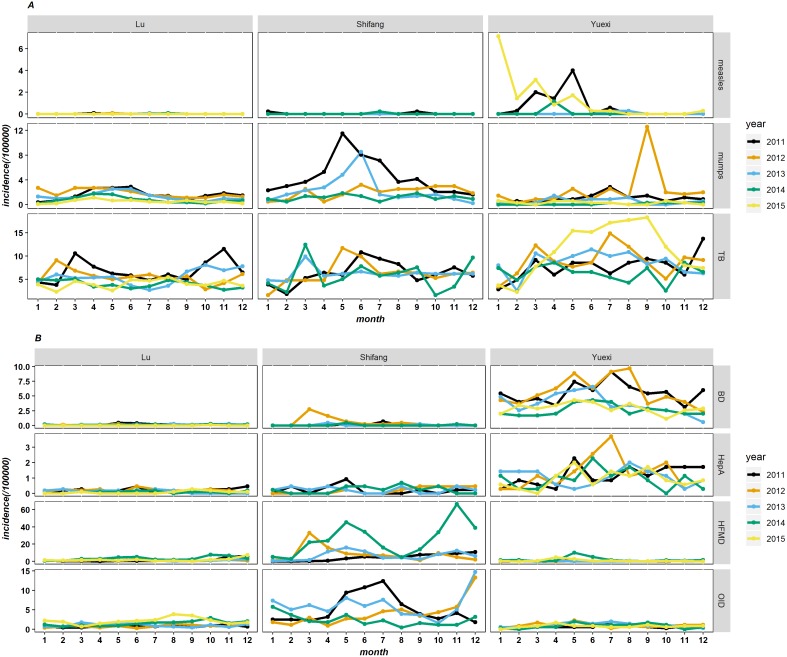
The monthly incidence rates of respiratory (A) and intestinal (B) infectious diseases of three counties in Sichuan, China, 2011–2015. TB, pulmonary tuberculosis; BD, bacillary dysentery; HepA, hepatitis A; HFMD, hand-foot-mouth disease; OID, other infectious diarrhea.

The incidence rates of measles in Yuexi was higher than that in other counties, and measles mainly occurred before June in 2011, 2014 and 2015. The incidence rates of mumps in Shifang showed upward trends from April to June in 2011 and 2013; in Yuexi, mumps broke out in September 2012, but there were no obvious seasonal trends in other years. In Shifang, the incidence rates of TB was higher from May to July in 2011 and 2012, and the incidence rates in March was higher than that in other months in 2013 and 2014; in Yuexi, the peak of TB occurred from April to October in 2015 (Fig. 4).

The incidence rates of bacterial dysentery and hepatitis A in Yuexi was higher than that in other countise.The incidence rates of bacterial dysentery in 2011 and 2012 showed upward trends before June, decreased in June, and then increased again in July and August; the incidence rates of hepatitis A was higher from May to July. The incidence rates of HFMD and other infectious diarrhea in Shifang was higher than that in other counties, and the incidence rates of HFMD in 2014 showed bimodal distribution, with incidence rates from April to June and from October to December that were higher than those in other months. For other infectious diarrhea, the incidence rates from May to July was higher in 2011 and 2013, and the incidence rates in December 2012 and 2013 was higher than that in other months (Fig. 4).

### Distributions of age and gender of RIDs and IIDs

**Figure 5:**
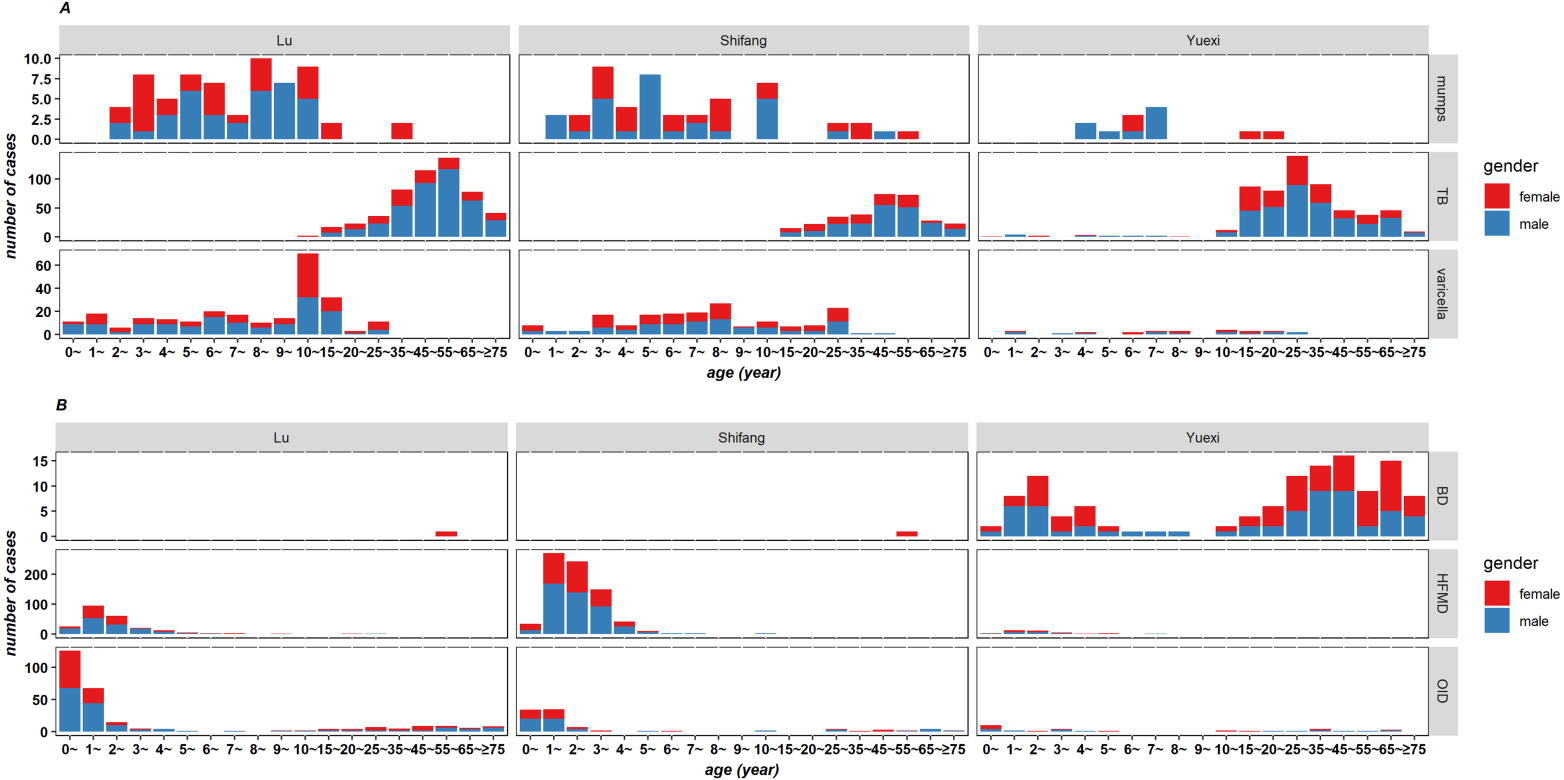
Age and gender distribution of respiratory (A) and intestinal (B) infectious diseases of three counties in Sichuan, China, 2015.

The age distribution of major RIDs and IIDs was similar in the three counties from 2011 to 2015 (Fig. 5 and Figs. S1–S4); therefore, the next analysis was based on the data of 2015. There was the largest number of TB cases in the 45∼65 age range in Lu and Shifang, and in Yuexi, the age range was 15∼45 years. There were the most cases of bacillary dysentery in the age range of 1∼5 years and at ages ≥15 years in Yuexi. HFMD cases mainly occurred in children under 5 years old, and the peak age range was 1∼3 years old. In Lu, Shifang and Yuexi, the male to female ratios of RIDs cases were 2.02:1, 1.61:1 and 1.59:1, respectively; and the ratios of IIDs were 1.31:1, 1.43:1 and 1.11:1, respectively.

### Influencing factors of RIDs

We established a linear regression model by taking the incidence rates of RIDs as the dependent variable (*y*) and the three PC scores as independent variables, and we obtained the following regression equation: *y* = 145.154 − 23.748PC1 − 43.085PC2. The standardized regression equation is as follows: *y* =  − 0.254PC1 − 0.496PC2. According to the PC score (Table 5), the standardized regression equation of the original variables was *y* = 0.011*Z*1 + 0.164*Z*2 + 0.179*Z*3 − 0.146*Z*4 − 0.090*Z*5 − 0.005*Z*6 − 0.019*Z*7 − 0.021*Z*8 + 0.150*Z*9 − 0.103*Z*10. The incidence rates of RIDs was positively associated with the proportion of individuals in the population younger than 5 and older than 60 years old, the ethnic minority population, and illiteracy and awareness of infectious disease knowledge. The incidence rates was negatively associated with per capita disposable income and the population proportion with correct drinking water habits, correct hand-washing habits, CPR skills, sanitary toilet use and awareness of emergency telephone availability. The top three influencing factors were the proportion of illiterate individuals, the ethnic minority population and the per capita disposable income.

**Table 5:** Results of principal component analysis of influencing factors in Sichuan, China.

	Standardized variables	Factor loading after rotation			Factor score coefficients		
		PC1	PC2	PC3	PC1	PC2	PC3
Proportion of population <5 years and >60 years	Z1	0.803	−0.161	0.050	0.547	−0.303	−0.195
Proportion of ethnic minorities population	Z2	−0.276	−0.857	−0.236	0.113	−0.388	0.037
Proportion of illiterate population	Z3	0.080	−0.838	−0.039	0.275	−0.501	0.087
Per capita disposable income	Z4	0.676	0.574	0.063	0.276	0.154	−0.270
Proportion of correct drinking habits population	Z5	0.621	0.575	0.378	0.135	0.113	0.022
Proportion of correct hand-washing habits population	Z6	0.559	0.346	0.563	0.091	−0.037	0.244
Proportion of population had the skill of CPR	Z7	0.453	0.448	0.590	−0.012	0.044	0.284
Proportion of population used sanitary toilet	Z8	0.692	0.275	0.406	0.248	−0.085	0.076
Awareness rate of infectious disease prevention and control knowledge	Z9	0.064	0.024	0.919	−0.269	−0.164	0.774
Awareness rate of emergency telephone	Z10	0.601	0.546	0.263	0.168	0.121	−0.066
Eigenvalue		2.931	2.819	1.951			
Contribution rate (%)		29.312	28.188	19.513			
Cumulative contribution rate (%)		29.312	57.500	77.013			

**Notes.**

PC principal component CPR cardiopulmonary resuscitation

## Discussion

In this research, we analysed the epidemic characteristics of RIDs and IIDs in three counties in Sichuan from 2011 to 2015. The main findings of this paper are the following: TB was the most prevalent RIDs in the three counties studied. The incidence rates of TB in Yuexi was higher than that in the other counties. HFMD and other infectious diarrhea were the major IIDs in Lu and Shifang; however, bacillary dysentery was the major IIDs in Yuexi. Moreover, the risk factors of RIDs included the proportion of illiterate individuals, the ethnic minority population and the per capita disposable income.

In 2014, the incidence rates of tuberculosis in China was 65.63/100,000. The incidence rates in Lu (49.75/100,000) was lower, the incidence rates in Yuexi (76.59/100,000) was higher, and the incidence rates in Shifang (69.89/100,000) was similar to the incidence rates in China. The incidence rates of measles in China in 2014 was 3.88/100,000; the incidence rates in the three counties were lower than that in China. In 2014, the incidence rates of pertussis in Yuexi (1.14/100,000) was higher than the incidence rates in China (0.25/100,000) (Zhen, Nian & Qiyan, 2018). From 2011 to 2015, the incidence rates of hepatitis A in China were 2.35/100,000, 1.81/100,000, 1.64/100,000, 1.92/100,000 and 1.66/100,000, and the incidence rates in Shifang and Yuexi were higher than those in China. From 2011 to 2015, the incidence rates of bacillary dysentery in Yuexi were higher than those in China, and the incidence rates in the other two counties were lower than the incidence rates in China (Bin & Ying, 2018).

From 2011 to 2015, the incidence rates of TB in Shifang remained stable, and Lu and Yuexi (except 2015) showed separate downward trends. The incidence rates of TB was the highest among RIDs. In 2015, the incidence rates of TB accounted for 62.40% of RIDs in Lu, 56.59% in Shifang, and 84.98% in Yuexi. In 2015, the incidence rates of TB in China was 63.42/100,000 (Guoqi et al., 2017). In 2015, the incidence rates of TB in Lu (49.29/100,000) was lower than the national level, and the incidence rates in Shifang (71.04/100,000) and Yuexi (161.75/100,000) were higher. These differences might be related to the fact that Sichuan province mainly relies on agriculture, meaning that 78% of the population lives in rural areas, and the rural economy, living conditions, education, and health care are relatively poor.

The incidence rates of TB in Yuexi was higher than that in other counties. According to the incidence rates ranking of TB in 2015, the top ten towns were all in Yuexi, and the incidences were higher than 200.00/100,000, of which the highest incidence rates of TB was in Matuo (1056.60/100,000). The per capita disposable income of Yuexi (median was 2822.50 Yuan) was significantly lower than that in the other counties, which indicated that the nutritional level of Yuexi residents might be poor. Undernutrition is an important risk factor for TB (Anuradha et al., 2018; Boelaert & Gordeuk, 2002). According to the global burden of associated risk factors, the attributable risk of undernutrition was estimated at 26.9% (Dheda, Barry & Maartens, 2016). Undernutrition can weaken the function of the immune system and activate latent TB infection, and the occurrence of TB may lead to increased metabolism and decreased appetite, which can aggravate malnutrition. Additionally, Yuexi is located in a high-altitude area and experienced more natural disasters, which were risk factors for TB incidence (Yang, Zhou & Pan, 2017).

HFMD was more prevalent in Shifang, and the incidences in 2014 and 2015 were higher than the national average of 139.78 per 100,000 (Zhuang et al., 2015). HFMD often occurs in children (Wang et al., 2014). Monthly average rainfall was a risk factor for HFMD (Wu et al., 2017), as moist air is conducive to the reproduction and transmission of enterovirus (Yang et al., 2018). Both Shifang and Yuexi had high annual precipitation; however, the incidence rates of HFMD in Shifang (174.50/100,000 in 2015) was significantly higher than that in Yuexi (10.29/100,000 in 2015). This difference might be related to population density and the proportion of population <5 and >60 years in Shifang, which were higher than those in Yuexi (530.44 persons/km^2^ VS 155.08 persons/km^2^ and 27.63% VS 19.66%). In 2014, the incidence rates of HFMD in Shifang reached 306.47/100,000, which was significantly higher than that in other years. This increase may be due to the high incidence rates of invisible infections of HFMD or the periodicity of the infectious disease, especially in childhood (Goldstein et al., 2017).

Note that bacterial diarrhea rarely occurred in Yixian and Shifang, while the incidence rates of bacterial diarrhea in Yuexi was more than 30.00/100,000. This difference might be due to the following reasons. First, Yuexi is among the high-risk regions of bacterial diarrhea, which share similar features: a. they are mountainous areas on the edge of the Tibetan Plateau; b. they have a warm climate and ample precipitation; c. they have an underdeveloped economy (Han et al., 2016). Second, the prevalence of unhygienic living habits was high (Kotloff et al., 2018); there were exceedingly few residents in Yuexi who drank safe drinking water and used sanitary toilets. Third, the education level of Yuexi residents was relatively low, and few residents had knowledge regarding the prevention and control of infectious diseases.

In this study, principal component regression was used to analyse the influencing factors of RIDs. We found that the incidence rates of RIDs was positively associated with the proportions of the ethnic minority population and the illiterate. The incidence rates of RIDs was negatively associated with per capita disposable income. These results were consistent with the findings reported in the literature (Lee et al., 2017; Yang, Zhou & Pan, 2017). In controlling infectious diseases, not only microbiological and environmental factors but also the impacts of population composition, economic and personal hygiene habits on diseases should be considered.

Some limitations should be noted in our research. First, the IIDs (like HFMD and other infectious diarrhea) were mainly concentrated in children, and our investigation of residents’ health behaviors was conducted in adults. Additionally, there was a great difference in the composition of IIDs among the three counties. The major IIDs in Yuexi was bacterial diarrhea, while in Lu and Shifang, the major IIDs were HFMD and other infectious diarrhea. Therefore, we did not analyse the influencing factors of IIDs. Second, the age distribution was analysed by the number of reported cases because the population and the incidence data for each age group in the three counties were not available.

## Conclusions

TB was the main RID in the three counties studied. The main IIDs in Lu and Shifang were HFMD and other infectious diarrhea, and in Yuexi, the main IID was bacillary dysentery. The incidence rates of TB, bacillary dysentery and hepatitis A in Matuo, Yuexi were the highest compared with other towns. The incidence rates of RIDs was associated with the population composition, the economy and personal hygiene habits.

##  Supplemental Information

10.7717/peerj.7341/supp-1 File S1 The diagnostic criteria for each infectious disease

10.7717/peerj.7341/supp-2 Table S1 Emergency monitoring capacity of Centers for Disease Control in three counties of Sichuan, China

10.7717/peerj.7341/supp-3 Table S2 Incidence rates of respiratory infectious diseases of three counties in Sichuan, China, 2011–2015

10.7717/peerj.7341/supp-4 Table S3 Incidence rates of intestinal infectious diseases of three counties in Sichuan, China, 2011–2015

10.7717/peerj.7341/supp-5 Figure S1 Age and gender distribution of respiratory and intestinal infectious diseases of three counties in Sichuan, China, 2011

10.7717/peerj.7341/supp-6 Figure S2 Age and gender distribution of respiratory and intestinal infectious diseases of three counties in Sichuan, China, 2012

10.7717/peerj.7341/supp-7 Figure S3 Age and gender distribution of respiratory and intestinal infectious diseases of three counties in Sichuan, China, 2013

10.7717/peerj.7341/supp-8 Figure S4 Age and gender distribution of respiratory and intestinal infectious diseases of three counties in Sichuan, China, 2014

10.7717/peerj.7341/supp-9 Data S1 Data for geographic map of three counties in Sichuan, China Data S1 applied for data analyses and preparation for Fig. 1.

10.7717/peerj.7341/supp-10 Data S2 Data for incidence rates of respiratory and intestinal infectious diseases, 2011–2015

10.7717/peerj.7341/supp-11 Data S3 Data for heat map of incidence rates of respiratory and intestinal infectious diseases, 2011–2015

10.7717/peerj.7341/supp-12 Data S4 Data for the monthly incidence rates of respiratory and intestinal infectious diseases, 2011–2015

10.7717/peerj.7341/supp-13 Data S5 Data for age and gender distribution of respiratory and intestinal infectious diseases, 2011–2015

10.7717/peerj.7341/supp-14 Data S6 Data for principal component analysis

## Additional Information and Declarations

### Data Availability

The following information was supplied regarding data availability: The raw measurements are available in the Supplemental Files. The raw data include incidence of RIDs and IIDs, and the descriptive of population composition, health related behaviors and awareness of towns.
