# Supplementary material for: Epidemiological analysis of respiratory and intestinal infectious diseases in three counties of Sichuan: the baseline survey of Disaster Mitigation Demonstration Area in western China
Source: PeerJ. 2019 Jul 23;7:e7341. doi: 10.7717/peerj.7341 (PMC6659668; doi:10.7717/peerj.7341)
Supplement: File S1 [file peerj-07-7341-s001.docx]

**The diagnostic criteria for each infectious diseases**

**Measles**

**Suspected cases:**

Patients (mostly children) have a fever, pharynx red and other upper respiratory catarrhal symptoms, photophobia, tears, conjunctival erythema and other acute conjunctivitis symptoms, fever for about 4 days, systemic skin erythema papules, and contact history with patients with gonorrhea 14 days ago.

**Confirmed cases:**

a. Koplik spots were seen in the buccal mucosa of the mouth.

b. Measles virus can be extracted from pharyngeal or conjunctival secretions.

c. Measles IgM antibody was detected in serum without measles vaccine in one month.

d. [Antibody titer](https://www.baidu.com/link?url=R3GRKKBOlxVvY8Y6sjtPDQfCJdFQLi8DxtFmo-zR17RBrTbeT0Eh093dy8xu8wGJCKE1ruKaLU522LaSLgYb2-Lzy1C8PLMuJIp8csHq6bf2Z0TavTB-a3PC8uHCcb5H&wd=&eqid=e3dbedcd001486f2000000035cae0dec) of measles IgG in convalescent serum was more than 4 times higher than that in an acute stage, or the antibody was negative in the acute stage and positive in the convalescent stage.

**Clinical diagnosis:** suspected cases plus one item.

**Laboratory confirmation:** suspected cases plus b or c or d items.

**Pulmonary tuberculosis (TB)**

**Suspected cases:** Where one of the following items is met:

a. Sputum tuberculosis test negative, chest X-ray suspected of active pulmonary tuberculosis;

b. The sputum tubercle bacillus test was negative, the chest X-ray examination showed the abnormal shadow, the patient had symptoms of tuberculosis such as cough, spitting, low fever, night sweat or did not absorb after 2-4 weeks of observation according to pneumonia treatment.

c. Children with strong positive tuberculin test (5 units, equivalent to 1:2000), accompanied by clinical symptoms of tuberculosis

**Confirmed cases:** Where one of the following items is met:

a. Sputum tuberculosis test positive (including smear or culture);

b. Sputum tuberculosis test negative,

c. The pathological diagnosis of pulmonary lesions was tuberculosis;

d. Suspected pulmonary tuberculosis, after clinical and X-ray follow-up, can exclude other pulmonary lesions;

e. Tuberculous pleurisy can be diagnosed by excluding pleural effusion caused by other causes.

**Scarlet fever**

**Suspected cases:**

fever, sore pharynx, skin hyperemia red dot rash or hyperemia miliary rash.

**Confirmed cases:**

a. Sudden fever, angina, raspberry tongue or bayberry tongue, pale mouth, skin folds at the red line (Papanicolaou line).

b. The rash appeared within 1-2 days after onset, and the skin was diffuse hyperemia and flushing. During the rash, pinpoint-sized scarlet skin rash was scattered, and the pressure faded. After 2-5 days, the rash subsided.

c. Within one week of rash abatement, the skin was desquamated or desquamated.

d. The total number of white blood cells and neutrophils in routine blood were increased.

e. Group A Streptococcus was isolated from throat swab or pus culture.

f. Detection of Group A Streptococcus by immunofluorescence with pharyngeal swab smear;

g. Erythema discoloration test was positive.

h. The polyvalent erythematoxin test was positive in the early stage of onset and negative in the recovery stage.

**Clinical diagnosis:** suspected cases plus d and a, or b, or c.

**Laboratory confirmation:** suspected cases plus e or f or g or h.

**Mumps**

**Suspected cases:**

Fever, chills, fatigue, loss of appetite, unilateral or bilateral non-suppurative parotid swelling pain or other salivary gland swelling pain after 1-2 days.

**Confirmed cases:**

a. Swelling and tenderness of parotid gland or other salivary glands are more obvious when eating acidic food. Swelling can be seen at the mouth of the parotid gland, the white blood cell count is normal or slightly low, and lymphocyte increases later.

b. There was a history of close contact with parotid patients within 8 to 30 days.

c. Mumps virus can be isolated from saliva.

d. Serum specific IgM antibody was positive.

e. The titer of serum IgG antibody in the convalescent stage was more than 4 times higher than that in the acute stage, or serum IgG antibody was positive in the convalescent stage.

**Clinical diagnosis:** suspected cases plus 1 reference 2.

**Laboratory confirmation:** suspected cases plus 3 or 4 or 5.

**Rubella**

**Suspected cases:**

Fever, red macular papules, enlargement of lymph nodes in the posterior ear or submandibular or cervical region, or joint pain.

**Confirmed cases:**

a. There was a clear contact history with rubella patients within 14-21 days.

b. Measles live vaccine has been vaccinated in eight years.

c. For the [peripheral blood](https://www.baidu.com/link?url=nmXcHKfk79blHqV10Qq0z2abFlksetRxLEDJhbMSnufL9PonxzSgC-cyBQERilC9_sESs0HM8_JKy9R1XZ5ywLNWMKoBVkZYS7XyF4G4-qN-jQ_QOhwhWdf1VjbmDXHy&wd=&eqid=ace61b95000176ec000000035caecc9a), the total number of white cells decreased and lymphocyte increased.

d. Rubella virus was isolated from pharyngeal swab specimens or urine or organ biopsy specimens.

e. Patient with serum rubella IgM antibody positive.

f. The titer of serum IgG antibody in the convalescent stage was more than 4 times higher than that in the acute stage, or serum IgG antibody was positive in the convalescent stage.

**Clinical diagnosis:** suspected cases plus a or a and b or a and c.

**Laboratory confirmation:** suspected cases plus d or e or f.

**Pertussis**

**Suspected cases:**

Those with a persistent paroxysmal spasmodic cough during the epidemic season.

**Confirmed cases:**

a. Close contact history with pertussis patient;

b. Significant increase of peripheral white blood cells and lymphocyte usually account for more than 50% of the total.

c. Haemophilus pertussis was isolated from sputum or throat of patients.

d. The serum antibodies in the convalescent stage were more than 4 times higher than those in the acute stage.

**Clinical diagnosis:** suspected cases plus 1 and 2.

**Laboratory confirmation:** suspected cases plus 3 or 4.

**Varicella**

**Clinical diagnosis:** consistent with a and b, or c

a. The history of varicella cases appeared in the close contact population within 14 days.

b. No other cases of rash

c. The rash first appeared on the scalp and trunk, then spread to the limbs, with the trunk being the most common. The rash is a red rash with itching at the beginning, then develops into a maculopapular rash, the surface of the rash forms a blister with a diameter of 1-4 mm. The blister fluid becomes turbid after several hours of transparency, scab formation after one day, and scab skin exfoliation after one week.

**Hepatitis A:**

**Suspected cases:**

a. About one month before the onset of the disease (2 to 6 weeks), the patient had been in contact with hepatitis A patients, or had been to work, travel and eat at the hepatitis A outbreak site or come directly from the epidemic site

b. Serum ALT level was increased.

c. Serum anti-HAV IgM antibody was positive.

d. The IgG titer of anti-HAV in two serum samples increased fourfold in the acute recovery phase.

e. 27 nm hepatitis A virus particles were observed in feces by immunoelectron microscopy.

**Clinical diagnosis:** suspected cases plus a or b items.

**Laboratory confirmation:** suspected cases plus any one of c, d, e.

**Hepatitis E (intestinal-transmitted non-A, non-B hepatitis):**

**Suspected cases:**

a. Had been exposed to hepatitis E two months before the onset of the disease, or to the hepatitis E outbreak site to work, travel, and eat, or dinner.

b. Serum ALT level was increased.

c. Serum anti-HEV IgM positive.

d. 30-32 nm virus particles were observed in feces by immunoelectron microscopy.

e. The cases did not conform to the infection of hepatitis A and B, CMV and EBV.

**Clinical diagnosis:** suspected cases plus two items b, e.

**Laboratory confirmation:** in line with clinical diagnosis plus c, d in term of a.

**Bacillary dysentery**

**Suspected cases:**

a. An acute episode of diarrhea (except for other causes of diarrhea), accompanied by fever, abdominal pain, purulent blood stool or mucus stool, tenderness in the left lower abdomen;

b. Stool microscopic examination of white blood cells (pus cells) per high (400 times) more than 15 visual fields, can be seen a small number of red blood cells

c. Shigella was positive in fecal bacteria culture.

**Clinical diagnosis:** a or b items.

**Laboratory diagnosis:** a or c items.

**Typhoid and paratyphoid fever**

**Suspected cases:**

In typhoid endemic areas, persistent fever lasted more than one week.

**Confirmed cases:**

a. Can not be excluded from other causes caused by persistent high fever (heat type is residual heat or relaxation heat), chilly, depression, anorexia, headache, anorexia, abdominal distension, skin can appear rose rash, spleen, relatively slow pulse.

b. Leukocytes and eosinophils decreased slightly at [peripheral blood](http://www.baidu.com/link?url=yaV3SkvM4KK7Y-psTCLlRiuUzp4OAs7hMx0sT0zJZRvZMn67jNhYgWaBYuiwtHMx6XzM2qn6FgHyvBvhah52KAbPKjydyvCt2WzCDM6wuh3O5gQ-N7SDXIKS383EMP9L).

c. Typhoid or paratyphoid bacilli were isolated from blood, bone marrow, urine and feces.

d. Serum specific antibody was positive. The agglutination titers of "O" antibodies were above 1:80, and those of "H", "A", "B" and "C" antibodies were above 1:160. Serum antibodies increased 4 -fold in acute and convalescent phase.

**Clinical diagnosis:** suspected cases plus a or b items.

**Laboratory confirmation:** suspected cases plus 3 or 4 items.

**Acute hemorrhagic conjunctivitis**

**Suspected cases:**

Acute eyelid redness, conjunctival congestion, bulbar conjunctival edema, bleeding and systemic symptoms are not obvious.

**Confirmed cases:**

a. There is an acute hemorrhagic conjunctivitis epidemic, contact with hemorrhagic conjunctivitis patients or a 24-hour history of exposure to swimming pool water or public towels.

b. Eye virus was positive and identified as EV70 or COXA24 variant or adenovirus.

c. Variant antigens of EV70 or COXA24 variants were found in eye secretions.

d. IgM or IgA antibodies in eye secretions against any of the above viruses were positive in blood.

e. The titer of IgG antibody of any of the above viruses in convalescent serum was more than 4 times higher than that in acute serum or the antibody was positive.

**Clinical diagnosis:** suspected cases plus a.

**Laboratory confirmation:** suspected cases plus b or c or d or e.

**Other infectious diarrhea**

**Clinical diagnosis:**

Stool tests except for diarrhea caused by cholera, dysentery, typhoid and paratyphoid infections, sparse or watery stools 3 or more times a day, loss of appetite, vomiting or non-vomiting, may be accompanied by fever, abdominal pain and general discomfort.

**Laboratory** **diagnosis:**

Other enteropathogenic bacteria or parasites or enteropathogenic viruses, viral antigens or specific nucleic acids were isolated from stools of diarrhea patients.

**Acute flaccid paralysis**

**Suspected cases:**

Any case of acute tardive paralysis that cannot be immediately identified as another cause.

**Confirmed cases:**

a. There is a history of contact with a confirmed poliomyelitis patient. The incubation period is 2 to 35 days (usually 7 to 14 days):

a) Fever, restlessness, hyperhidrosis, neck and back stiffness and gastrocnemius tenderness, etc.

b) After fever recession, muscle tension of body or limbs weakened, deep tendon reflex weakened or disappeared, and asymmetric (or bilateral) flaccid paralysis, no sensory disorder, muscle atrophy in the later stage.

b. 60 days after the onset of the disease, there is still residual flaccid paralysis

c. The virus was isolated from feces, cerebrospinal fluid and pharynx and identified as poliovirus.

d. Virus isolated from brain or spinal cord tissue and identified as poliovirus

e. No polio vaccine was given within 1 month, but specific IgM antibodies were found in cerebrospinal fluid or blood.

f. The titer of neutralizing antibody or specific IgG antibody in serum of convalescent patients was more than 4 times higher than that in acute phase.

**Amebic dysentery**

**Suspected cases:**

Mild onset, abdominal pain, diarrhea, dark red stool, with blood, pus or mucus, or pasty, with a fishy odor.

**Confirmed cases:**

a. Fecal examination revealed cysts or small trophozoites.

b. Fecal examination revealed amoeba macro trophozoites.

c. Sigmoid colonoscopy revealed that amoeba trophozoites were found in the intestinal tissue.

**Clinical diagnosis:** suspected cases plus an item

**Laboratory confirmation:** suspected cases plus b or c items

**Hand-foot-mouth disease (HFMD)**

**Clinical diagnosis:**

a. History of Epidemiology: It is common in preschool children and infants. The prevalence of the HFMD among the local and young people and the surrounding population is the direct or indirect contact history of the children with HFMD before the onset of the epidemic.

b. Clinical manifestation

It is in accordance with the above-mentioned clinical In rare cases, the rash is not typical, and some cases only appear as encephalitis or meningitis, and the diagnosis needs to be combined with the pathogenic or serological test results.

**Confirmed cases:**

On the basis of clinical diagnosis, one of the following can be confirmed:

a. Specific nucleic acid test positive for enterovirus (CV-A16, EV-A71,etc.)

b. The enterovirus was identified as CV-A16, EV-A71 or other enteroviruses that could cause HFMD.

c. IgM antibody of the serum-associated virus was positive in the acute phase.

d. The neutralizing antibody of serum-associated enterovirus in convalescence was 4 times higher than that in an acute stage.
