# Supplementary material for: Epidemiological analysis of respiratory and intestinal infectious diseases in three counties of Sichuan: the baseline survey of Disaster Mitigation Demonstration Area in western China
Source: PeerJ. 2019 Jul 23;7:e7341. doi: 10.7717/peerj.7341 (PMC6659668; doi:10.7717/peerj.7341)
Supplement: Table S1 [file peerj-07-7341-s002.docx]

**Table S1 Emergency monitoring capacity of Centers for Disease Control in three counties of Sichuan, China**

|  | Lu | Shifang | Yuexi |
| --- | --- | --- | --- |
| Number of water examination items |  |  |  |
| General test | 34 | 42 | 7 |
| Emergency test | 2 | 7 | 7 |
| Number of pathogenic microorganism examination items |  |  |  |
| General test | 6 | 10 | 3 |
| Emergency test | 6 | 2 | 3 |
