# Supplementary material for: Epidemiological analysis of respiratory and intestinal infectious diseases in three counties of Sichuan: the baseline survey of Disaster Mitigation Demonstration Area in western China
Source: PeerJ. 2019 Jul 23;7:e7341. doi: 10.7717/peerj.7341 (PMC6659668; doi:10.7717/peerj.7341)
Supplement: Table S2 [file peerj-07-7341-s003.docx]

**Table S2** **Incidence** **rates of respiratory infectious diseases of three counties in Sichuan, China, 2011-2015**

|  | Year | Measles | |  | Pulmonary tuberculosis | |  | Scarlet fever | |  | Mumps | |  | Rubella | |  | Varicella | |  | Pertussis | |  | Total | |
| --- | --- | --- | --- | --- | --- | --- | --- | --- | --- | --- | --- | --- | --- | --- | --- | --- | --- | --- | --- | --- | --- | --- | --- | --- |
|  |  | n | /100,000 |  | n | /100,000 |  | n | /100,000 |  | n | /100,000 |  | n | /100,000 |  | n | /100,000 |  | n | /100,000 |  | n | /100,000 |
| Lu | 2011 | 1 | 0.09 |  | 883 | 81.96 |  | 2 | 0.19 |  | 206 | 19.12 |  | 3 | 0.28 |  | 409 | 37.97 |  | 0 | 0.00 |  | 1504 | 139.61 |
|  | 2012 | 1 | 0.09 |  | 731 | 67.85 |  | 6 | 0.56 |  | 239 | 22.19 |  | 26 | 2.41 |  | 281 | 26.08 |  | 0 | 0.00 |  | 1284 | 119.19 |
|  | 2013 | 1 | 0.09 |  | 738 | 68.50 |  | 6 | 0.56 |  | 171 | 15.87 |  | 1 | 0.09 |  | 280 | 25.99 |  | 0 | 0.00 |  | 1197 | 111.11 |
|  | 2014 | 1 | 0.09 |  | 536 | 49.75 |  | 3 | 0.28 |  | 99 | 9.19 |  | 0 | 0.00 |  | 236 | 21.91 |  | 0 | 0.00 |  | 875 | 81.22 |
|  | 2015 | 0 | 0.00 |  | 531 | 49.29 |  | 1 | 0.09 |  | 65 | 6.03 |  | 0 | 0.00 |  | 250 | 23.21 |  | 4 | 0.37 |  | 851 | 78.99 |
| Shifang | 2011 | 2 | 0.46 |  | 334 | 76.79 |  | 3 | 0.69 |  | 240 | 55.18 |  | 5 | 1.15 |  | 98 | 22.53 |  | 0 | 0.00 |  | 682 | 156.80 |
|  | 2012 | 0 | 0.00 |  | 323 | 74.26 |  | 3 | 0.69 |  | 104 | 23.91 |  | 4 | 0.92 |  | 207 | 47.59 |  | 0 | 0.00 |  | 641 | 147.37 |
|  | 2013 | 0 | 0.00 |  | 323 | 74.26 |  | 9 | 2.07 |  | 114 | 26.21 |  | 5 | 1.15 |  | 254 | 58.40 |  | 0 | 0.00 |  | 705 | 162.08 |
|  | 2014 | 1 | 0.23 |  | 304 | 69.89 |  | 3 | 0.69 |  | 60 | 13.79 |  | 0 | 0.00 |  | 144 | 33.11 |  | 0 | 0.00 |  | 512 | 117.71 |
|  | 2015 | 5 | 1.15 |  | 309 | 71.04 |  | 2 | 0.46 |  | 51 | 11.73 |  | 1 | 0.23 |  | 178 | 40.92 |  | 0 | 0.00 |  | 546 | 125.53 |
| Yuexi | 2011 | 29 | 8.29 |  | 422 | 120.60 |  | 0 | 0.00 |  | 41 | 11.72 |  | 34 | 9.72 |  | 27 | 7.72 |  | 1 | 0.29 |  | 554 | 158.32 |
|  | 2012 | 0 | 0.00 |  | 393 | 112.31 |  | 1 | 0.29 |  | 101 | 28.86 |  | 4 | 1.14 |  | 33 | 9.43 |  | 6 | 1.71 |  | 538 | 153.75 |
|  | 2013 | 2 | 0.57 |  | 393 | 112.31 |  | 1 | 0.29 |  | 22 | 6.29 |  | 0 | 0.00 |  | 43 | 12.29 |  | 1 | 0.29 |  | 462 | 132.03 |
|  | 2014 | 5 | 1.43 |  | 268 | 76.59 |  | 0 | 0.00 |  | 5 | 1.43 |  | 3 | 0.86 |  | 17 | 4.86 |  | 4 | 1.14 |  | 302 | 86.30 |
|  | 2015 | 53 | 15.15 |  | 566 | 161.75 |  | 2 | 0.57 |  | 12 | 3.43 |  | 3 | 0.86 |  | 26 | 7.43 |  | 4 | 1.14 |  | 666 | 190.32 |
