# Supplementary material for: Epidemiological analysis of respiratory and intestinal infectious diseases in three counties of Sichuan: the baseline survey of Disaster Mitigation Demonstration Area in western China
Source: PeerJ. 2019 Jul 23;7:e7341. doi: 10.7717/peerj.7341 (PMC6659668; doi:10.7717/peerj.7341)
Supplement: Table S3 [file peerj-07-7341-s004.docx]

**Table S3 Incidence rates of intestinal infectious diseases of three counties in Sichuan, China, 2011-2015**

|  | year | Hepatitis A | |  | Hepatitis E | |  | Bacillary dysentery | |  | Typhoid and paratyphoid | |  | Acute hemorrhagic conjunctivitis | |  | Other infectious diarrhea | |  | Hand-foot-mouth disease | |  | AFP | |  | Amebic dysentery | |  | Total | |
| --- | --- | --- | --- | --- | --- | --- | --- | --- | --- | --- | --- | --- | --- | --- | --- | --- | --- | --- | --- | --- | --- | --- | --- | --- | --- | --- | --- | --- | --- | --- |
|  |  | n | /100,000 |  | n | /100,000 |  | n | /100,000 |  | n | /100,000 |  | n | /100,000 |  | n | /100,000 |  | n | /100,000 |  | n | /100,000 |  | n | /100,000 |  | n | /100,000 |
| Lu | 2011 | 27 | 2.51 |  | 34 | 3.16 |  | 20 | 1.86 |  | 0 | 0.00 |  | 15 | 1.39 |  | 102 | 9.47 |  | 189 | 17.54 |  | 2 | 0.19 |  | 0 | 0.00 |  | 389 | 36.11 |
|  | 2012 | 26 | 2.41 |  | 27 | 2.51 |  | 10 | 0.93 |  | 0 | 0.00 |  | 17 | 1.58 |  | 106 | 9.84 |  | 218 | 20.24 |  | 5 | 0.46 |  | 0 | 0.00 |  | 409 | 37.97 |
|  | 2013 | 15 | 1.39 |  | 11 | 1.02 |  | 13 | 1.21 |  | 3 | 0.28 |  | 29 | 2.69 |  | 132 | 12.25 |  | 147 | 13.65 |  | 4 | 0.37 |  | 0 | 0.00 |  | 354 | 32.86 |
|  | 2014 | 11 | 1.02 |  | 18 | 1.67 |  | 15 | 1.39 |  | 1 | 0.09 |  | 24 | 2.23 |  | 194 | 18.01 |  | 439 | 40.75 |  | 5 | 0.46 |  | 0 | 0.00 |  | 707 | 65.63 |
|  | 2015 | 9 | 0.84 |  | 15 | 1.39 |  | 1 | 0.09 |  | 2 | 0.19 |  | 23 | 2.13 |  | 276 | 25.62 |  | 229 | 21.26 |  | 2 | 0.19 |  | 0 | 0.00 |  | 557 | 51.70 |
| Shifang | 2011 | 11 | 2.53 |  | 1 | 0.23 |  | 5 | 1.15 |  | 0 | 0.00 |  | 14 | 3.22 |  | 277 | 63.68 |  | 237 | 54.49 |  | 0 | 0.00 |  | 0 | 0.00 |  | 545 | 125.30 |
|  | 2012 | 10 | 2.30 |  | 4 | 0.92 |  | 27 | 6.21 |  | 0 | 0.00 |  | 11 | 2.53 |  | 214 | 49.20 |  | 422 | 97.02 |  | 1 | 0.23 |  | 0 | 0.00 |  | 689 | 158.41 |
|  | 2013 | 10 | 2.30 |  | 5 | 1.15 |  | 2 | 0.46 |  | 0 | 0.00 |  | 14 | 3.22 |  | 317 | 72.88 |  | 344 | 79.09 |  | 1 | 0.23 |  | 0 | 0.00 |  | 693 | 159.33 |
|  | 2014 | 12 | 2.76 |  | 1 | 0.23 |  | 3 | 0.69 |  | 0 | 0.00 |  | 12 | 2.76 |  | 121 | 27.82 |  | 1333 | 306.47 |  | 1 | 0.23 |  | 0 | 0.00 |  | 1483 | 340.95 |
|  | 2015 | 21 | 4.83 |  | 3 | 0.69 |  | 1 | 0.23 |  | 0 | 0.00 |  | 12 | 2.76 |  | 99 | 22.76 |  | 759 | 174.50 |  | 1 | 0.23 |  | 0 | 0.00 |  | 896 | 206.00 |
| Yuexi | 2011 | 48 | 13.72 |  | 3 | 0.86 |  | 228 | 65.16 |  | 3 | 0.86 |  | 0 | 0.00 |  | 32 | 9.14 |  | 10 | 2.86 |  | 0 | 0.00 |  | 0 | 0.00 |  | 324 | 92.59 |
|  | 2012 | 55 | 15.72 |  | 3 | 0.86 |  | 239 | 68.30 |  | 1 | 0.29 |  | 0 | 0.00 |  | 44 | 12.57 |  | 11 | 3.14 |  | 1 | 0.29 |  | 0 | 0.00 |  | 354 | 101.16 |
|  | 2013 | 44 | 12.57 |  | 0 | 0.00 |  | 153 | 43.72 |  | 1 | 0.29 |  | 0 | 0.00 |  | 46 | 13.15 |  | 7 | 2.00 |  | 1 | 0.29 |  | 2 | 0.57 |  | 254 | 72.59 |
|  | 2014 | 39 | 11.15 |  | 1 | 0.29 |  | 107 | 30.58 |  | 1 | 0.29 |  | 0 | 0.00 |  | 36 | 10.29 |  | 85 | 24.29 |  | 1 | 0.29 |  | 3 | 0.86 |  | 273 | 78.02 |
|  | 2015 | 38 | 10.86 |  | 3 | 0.86 |  | 123 | 35.15 |  | 3 | 0.86 |  | 0 | 0.00 |  | 33 | 9.43 |  | 36 | 10.29 |  | 3 | 0.86 |  | 3 | 0.86 |  | 242 | 69.16 |
| AFP, acute flaccid paralysis | | | | | | | | | | | | | | | | | | | | | | | | | | | | | | |
