# Supplementary figures and images for: Epidemiological analysis of respiratory and intestinal infectious diseases in three counties of Sichuan: the baseline survey of Disaster Mitigation Demonstration Area in western China
Source: PeerJ. 2019 Jul 23;7:e7341. doi: 10.7717/peerj.7341 (PMC6659668; doi:10.7717/peerj.7341)

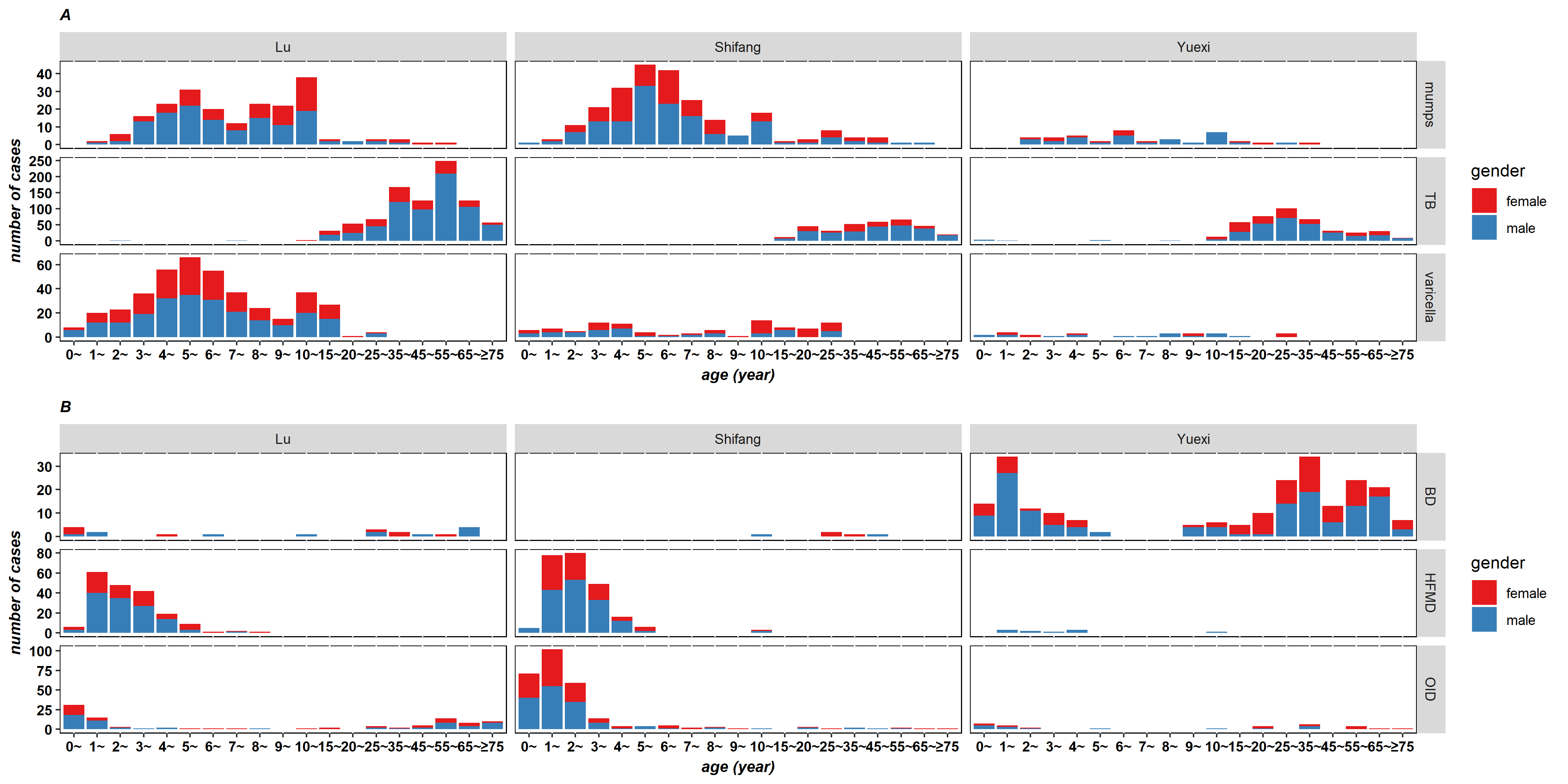

Supplement: Figure S1 [file peerj-07-7341-s005.png]

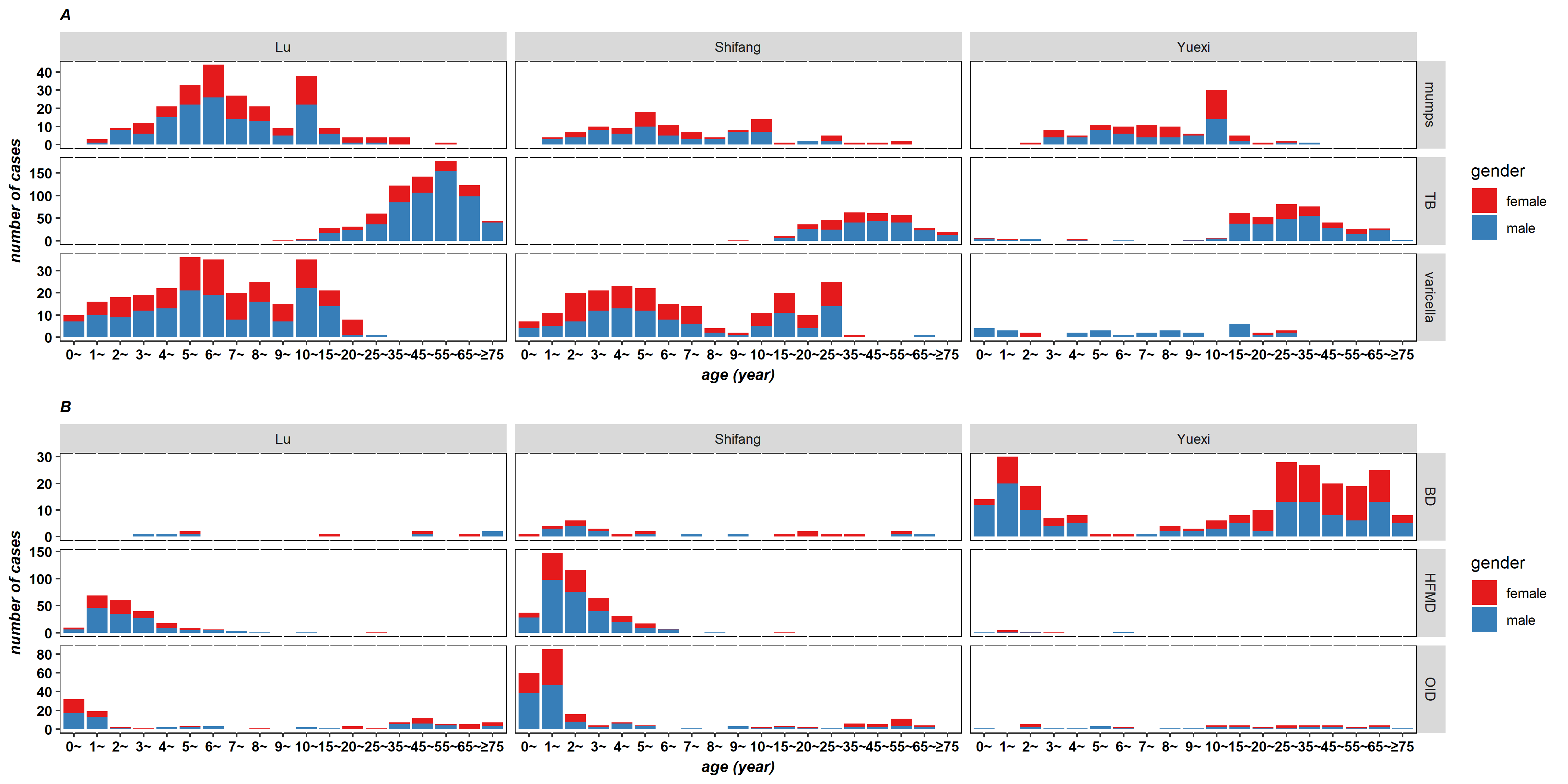

Supplement: Figure S2 [file peerj-07-7341-s006.png]

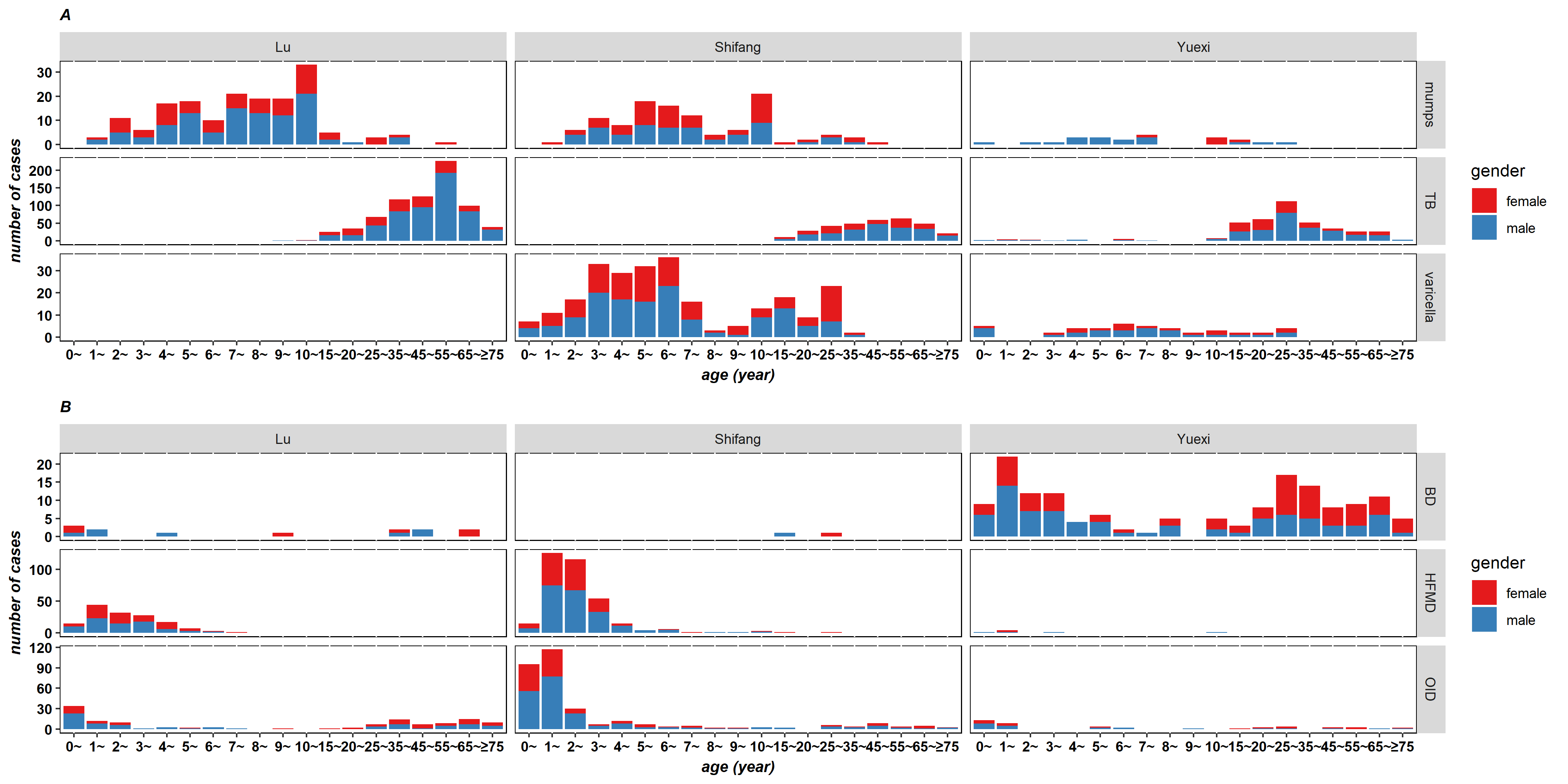

Supplement: Figure S3 [file peerj-07-7341-s007.png]

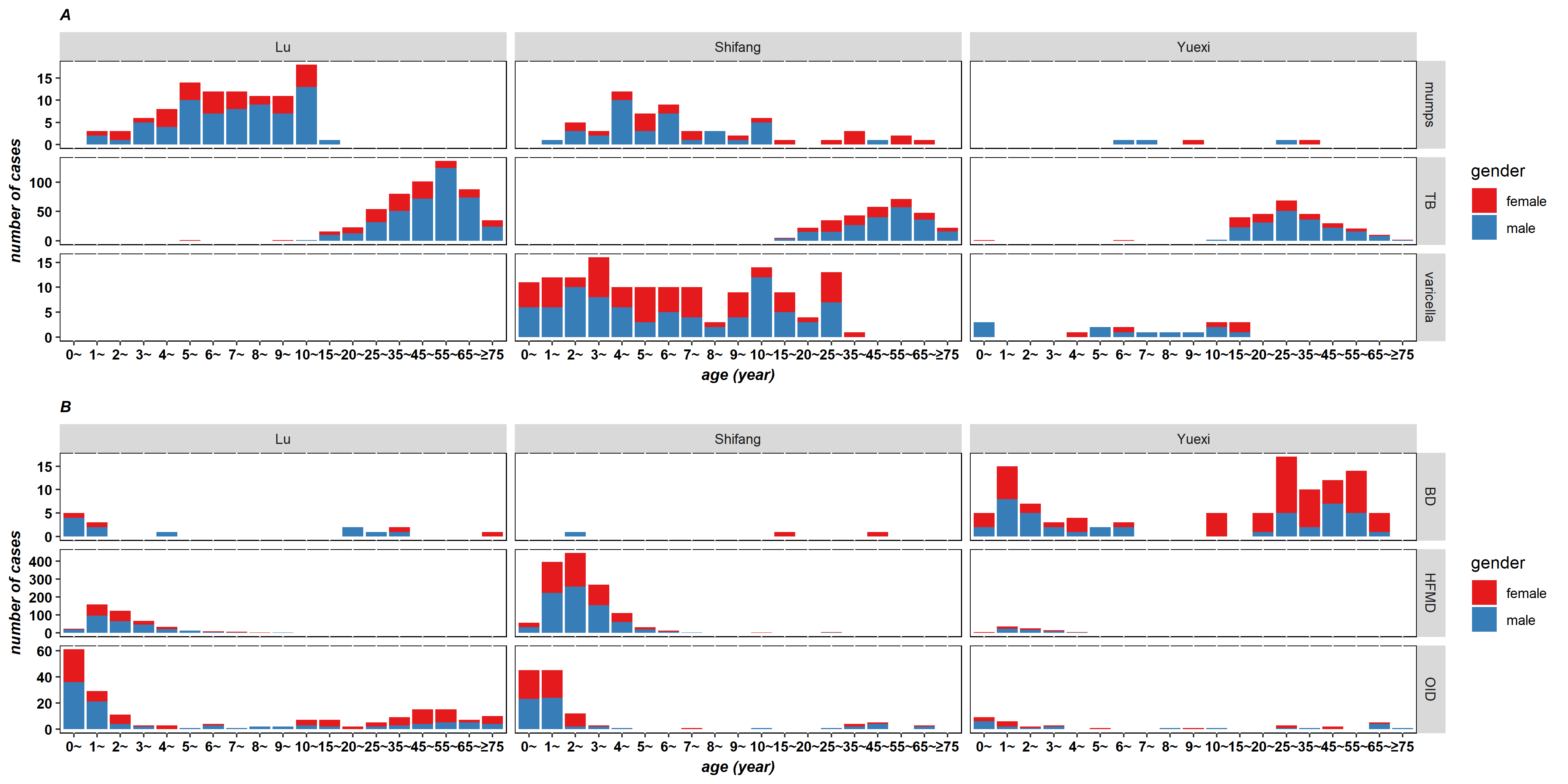

Supplement: Figure S4 [file peerj-07-7341-s008.png]
